# Translation and evaluation of the Perinatal Grief Intensity Scale in Kenya and Uganda

**DOI:** 10.1186/s12884-026-09394-6

**Published:** 2026-06-08

**Authors:** Allen Nabisere, Sabina Wakasiaka, James Dodd, Lucie Byrne-Davis, Maia Lesosky, Tina Lavender, Tracey A Mills, Elizabeth Ayebare

**Affiliations:** 1 School of Public Health, College of Health Sciences, Makerere University, P.O Box 7072, Kampala, Uganda; 2 University of Nairobi, Nairobi, Kenya; 3 Liverpool School of Tropical Medicine, Liverpool, UK; 4 The University of Manchester, Manchester, UK; 5 National Heart and Lung Institute, Imperial College London, London, UK; 6 Department of Nursing, College of Health Sciences, Makerere University, Kampala, Uganda

**Keywords:** Stillbirth, Neonatal death, Bereavement, Perinatal Grief Intensity Scale, Translation and evaluation, Sub-Saharan Africa

## Abstract

**Background and Aim:**

Stillbirth or neonatal death are distressing life experiences for parents. In many sub-Saharan African countries, bereavement care and support are inadequate. Context-appropriate interventions are needed to improve perinatal bereavement care. To assess whether these interventions work, reliable assessment tools are required. This study aimed to translate and evaluate the Perinatal Grief Intensity Scale [PGIS] for use among bereaved women in Kenya and Uganda.

**Methods:**

A mixed-methods study, involving cross cultural translation, adaptation and psychometric evaluation in Kenya and Uganda. The PGIS was translated into Kiswahili and Luganda with input from community engagement groups and stakeholders. Pilot-testing was conducted in 2 stages; ‘Think aloud’ interviews (*n* = 16) and test-retest (*n* = 20). The finalised tools were tested with 160 women who had experienced stillbirth or neonatal death, 20 in the test-retest phase and 140 in wider testing. Analyses included test-retest reliability, internal consistency and exploratory associations with participant characteristics using single variable linear regression.

**Results:**

Agreed translations of the PGIS in Kiswahili and Luganda were produced. In pilot testing, test-retest consistency was high with an intraclass correlation coefficient of 0.840 (95% CI, 0.590–0.937). Wider testing demonstrated acceptable internal consistency, Cronbach’s alpha of the total PGIS score was 0.737, confirming overall reliability, with similar results in the pilot testing and wider testing subgroups. Single variable linear regression showed that PGIS scores were higher for women in Uganda (coefficient 0.123, 95% Confidence Interval (CI) 0.042 to 0.205, p value = 0.003) compared to Kenya, and higher for caesarean births (coefficient 0.113, 95% CI 0.014 to 0.211, p value = 0.025) compared to vaginal births.

**Conclusion:**

Preliminary evaluation of the Kiswahili and Luganda versions of the PGIS demonstrated good reliability for use in Kenya and Uganda. The tools have potential for future use in research to assess the impact of bereavement care interventions and in clinical practice to identify women who would benefit from increased bereavement support. Further assessments, including factor analyses and predictive performance are required to establish validity in this context.

**Supplementary Information:**

The online version contains supplementary material available at 10.1186/s12884-026-09394-6.

## Introduction

The death of a baby before, during or shortly after birth is a profound tragedy for parents [1]. Although grief is a normal experience after the perinatal death, prolonged and intense responses can impair parent’s wellbeing [2] and are associated with psychological complications including severe anxiety, depression and post-traumatic stress [3]. Therefore, monitoring of the psychological status following a perinatal death is important for early detection and intervention to address distress [4, 5]. Health workers play a crucial in supporting normal grieving processes and reducing negative health and social outcomes after the death of a baby [6]. However, across settings, including sub-Saharan Africa which bears a disproportionate burden of global perinatal deaths, many parents do not receive the care and psychological support that they need [7].

Improving perinatal bereavement support has been identified as a priority for global action in maternal and newborn care [8]. However, few interventions have been robustly evaluated for effectiveness in supporting parent’s coping after the death of a baby [9]. A significant challenge is to identify and objectively measure relevant outcomes, which requires culturally appropriate and reliable tools. To address this, contextual adaptation of psychological instruments is important as it ensures that tools respect the unique values, languages, and social realities of a particular setting [10]. Several tools have been developed and used to assess grief after stillbirth or neonatal death [11], of these, the Perinatal Grief Scale ( PGS) and Perinatal Greif Intensity Scale have been the most widely used in research [11]. The PGS is a 33-item instrument developed and validated to identify intense grief reactions after perinatal loss [12]. Although the PGS has been translated and validated across several high and upper middle-income countries [13], it has been criticised for relatively high respondent burden which may impact usefulness in some settings [14]. The Perinatal Grief Intensity Scale (PGIS), a 14-item instrument was also developed to assess grief intensity in women after miscarriage, stillbirth or neonatal death [15]. It comprises three subscales ‘Reality’ assessing the perceived reality of pregnancy and the baby; ‘Confront Others’ assessing maternal ability to ask for support with needs and tackle unsupportive behaviour from significant others, and ‘Congruence’ focusing on acceptability of the current loss experience to the woman. When compared with the PGS, the PGIS demonstrated comparable reliability, validity, and performance in identifying intense grief [16]. It has fewer items, and easier to score than the PGS, and therefore may be suitable for low and middle income settings where literacy levels are variable [11]. However, this tool has not been translated or evaluated for use in sub–Saharan African settings, including Uganda and Kenya. Therefore, this study aimed to translate the PGIS into Kiswahili and Luganda (local languages) and evaluate use with women who had experienced a perinatal death in Kenya and Uganda.

## Materials and methods

### Study design

**Fig. 1:**
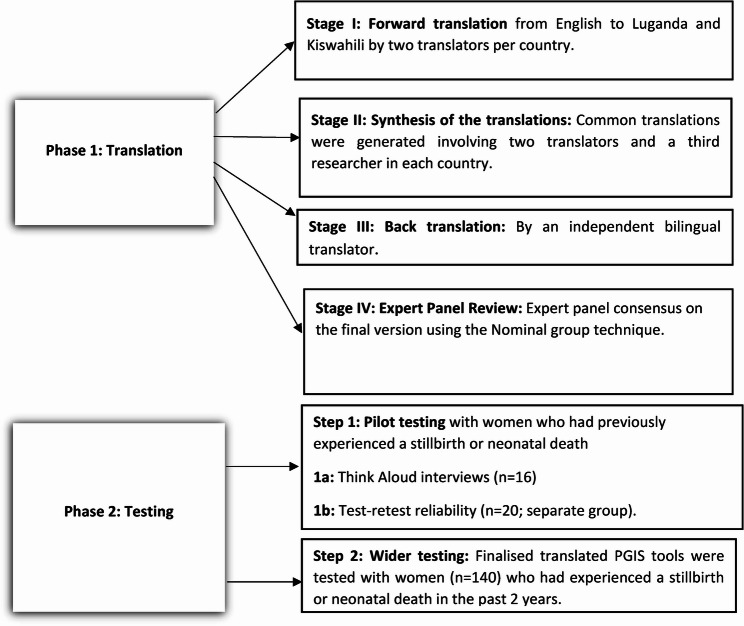
The translation and evaluation process

A two-phase, pragmatic, mixed-methods study was conducted from February 2022 to February 2023. The study followed the approach to cross-cultural translation and adaptation of self-report and study instruments described by Shrestha (Fig. 1) [11]. In this study, we translated and evaluated the Perinatal Grief Intensity Scale, an instrument originally developed in the United States for use in the clinical setting to measure grief intensity among women who have experienced a perinatal death and predict the need for follow-up [15, 16].

#### Phase one: Translation

##### Stage 1

Forward translation of the PGIS from English (source language) to Kiswahili and Luganda (target languages) was conducted simultaneously by a professional translator and a bilingual researcher with experience of maternity care and terminology.

##### Stage II 

A meeting involving the translators and an additional researcher was then held, to synthesise and generate agreed common translations in Luganda and Kiswahili.

##### Stage III

The common translations were then back-translated to English for accuracy, by a third bilingual translator, not previously connected to the study.

##### Stage IV

An expert panel meeting was held in each country to review the translations and agree the final versions for pretesting. The panel members; fifteen in Uganda and eight in Kenya, were recruited from the existing country stakeholder networks of the NIHR Global Health Research Unit on the Prevention and Management of Stillbirth and Neonatal death in sub-Saharan Africa and South Asia. Panels included midwives and obstetricians, methodologists, linguists, Community Engagement and Involvement (CEI) representatives and the research team including all the translators. A consensus approach, drawing on the nominal group technique, facilitated equal participation and timely agreement [11]. Following an overview of the research, panel members reviewed the translations and source documents, independently. Feedback for each item was shared in a ‘round robin’ manner, followed by a panel discussion on the appropriateness of the translations. Agreement on the final translation for each item was reached by voting where necessary; inclusion was determined by a majority vote among the panel members.

#### Phase two: Testing

##### Sample and recruitment

Testing was conducted in two stages; pilot and wider testing. Recruitment and data collection were conducted by trained research assistants (midwifery or nursing background), participants were women who had experienced a stillbirth (antenatal or Intrapartum fetal death ≥ 28 weeks’ gestation) or neonatal death (live birth at any gestation followed by death up to 28 days after birth).

In the pilot test, 36 women (16 for think aloud interviews and 20 for the test-retest) were recruited from existing and previous Community Engagement and Involvement (CEI) groups of the NIHR Global Health Research Unit on Prevention and Management of Stillbirths and Neonatal Deaths in Uganda and Kenya, who were not previously involved in the translation process.

For the wider test, an additional 140 women who had experienced stillbirth or neonatal death at 2 maternity facilities and surrounding communities in Uganda and Kenya, within the previous 2 years were recruited. Eligible women were identified and approached via the clinical care team before discharge from the hospital or a community health worker. Those who were interested in participating, completed a consent-to-contact form and were contacted by the research assistant not earlier than two weeks after hospital discharge. Women who were pregnant at the time of recruitment or who had a multiple birth where one baby was still living were ineligible to participate.

##### Data collection

Data were collected at a venue of the participant’s preference, with due regard for researcher safety. An investigator-designed questionnaire was used to capture demographic, birth and postnatal characteristics prior to completion of the translated PGIS questionnaire. In the wider testing phase, women completed the translated PGIS questionnaires between 8 weeks and 24 months post-birth.

#### Pilot testing

##### Stage 1

Ease of understanding, experiences of completion, face and content validity were assessed using cognitive ‘think aloud’ interviews, a method designed to capture participants’ thoughts and reactions while completing a particular task [11]. Sixteen women (N=8 per country) completed the tool in the presence of the researcher and were asked to verbalise thoughts as they responded to the items, the only prompts used were ‘think aloud’. Sample size was based on an estimate of data adequacy [11, 17, 18]. The interviews were digitally audio-recorded, with consent, and transcribed verbatim. Data informed amendments to the tool before stage 2.

##### Stage 2

The amended tools were then administered to an independent group of 20 women not previously recruited to the study (10 in each country) on two occasions, one week apart to assess reliability over time [19], following recommendations for a minimum interval of 7 days to assess consistency [20].

##### Wider Testing

The translated and amended PGIS tools were then administered to an additional cross-sectional sample of 140 women (70 per country). This resulted in a total sample of 160 participants completing the finalised tool, including the 20 women from the test-retest phase. This sample was based on a participant to item ratio of at least 10:1, being sufficient to allow planned analysis of floor and ceiling effects and reliability [21].

### Data management and analysis

Reports of the translation processes were compiled and used to modify the items in the translations. The think-aloud interview transcripts were read and re-read to achieve familiarisation and then analysed by descriptive content analysis, with an aim of identifying words or phrases which were perceived as unclear, inappropriate, or confusing.

Demographic data were collected on paper questionnaires, de-identified and entered into a password-protected REDCap database, and analysed descriptively using Stata 17 [22]. An Intraclass Correlation Coefficient (ICC) was calculated to assess external consistency of the translated questionnaires across two time-points, with Kappa values for individual components to assess agreement. Country stratified and overall estimates of PGIS average scores, and PGIS variance in scores were calculated. The mean PGIS score was computed by averaging scores for components for reality (questions 1–6), confront others (questions 7–10), congruence (questions 11–14) subscales, and then using the formula $\mathrm{PGIS}=\mathrm{3.08}+\left(\mathrm{0.41}\times\mathrm{reality}\right)-\left(\mathrm{0.2}\times\mathrm{confront}\right)-\left(\mathrm{0.15}\times\mathrm{congruence}\right)$. Note that components are scored from 1 = strongly agree to 4 = strongly disagree except for components 4 and 5 where scoring is reversed. A higher total PGIS score is indicative of more intense grief, a cut off score of > 3.53 is associated with high-intensity grief in studies with women in high income countries [16]. The maximum possible score is 4.37 [23]. Exploratory analyses were undertaken to understand difference between countries (descriptive summaries) and variation in score (univariable linear regression analyses). Cronbach’s alpha was computed to quantify internal consistency. Model assumptions for linear regression included approximately normally distributed conditional errors, a linear mean structure, homoscedasticity, and independence of observations. Analyses were not intended to be confirmatory, no formal correction for multiple testing was applied, and results should be interpreted accordingly. There were no missing data for the outcome (Perinatal Grief Intensity) or independent variables.

## Results

### Translation

During forward translation, the translators identified some words with no direct equivalents in Kiswahili or Luganda; ‘scale/subscale’, ‘reality’, ‘congruence’, ‘personality’ ‘perinatal’ and ‘Grief Scale’. Following agreement, these were translated using descriptive phrases or approximations. No discrepancies between the back translations and the original versions were identified in either country. Expert panel meetings reached consensus that the translations were correct. Some words and phrases were identified as needing further minor clarification to reflect the original intended meaning, in the local context. However, extensive modifications of items in the final translations were not required and the panel confirmed that no conceptual changes were made which were likely to have affected the constructs being assessed. Adjustments made for Kiswahili and Luganda are summarised in supplementary Tables S1 and S2, respectively.

### Think aloud interviews

Sixteen women participated in the think-aloud interviews, ranging from 15 to 48 min in duration. Analysis highlighted that items 2 and 3 of the Luganda translation had some similarity and were difficult to differentiate, a few grammatical and spelling errors were also identified. Other items were felt to be understandable. Minor revisions made to the Luganda tool are shown in supplementary Table S3. No changes were made to the Kiswahili tool at this point.

### Test -retest results

Twenty women participated in the Test-retest phase (supplementary table S4). The median age was 31 (IQR= (26.5–36) and median time since the death of the baby was 25.4 months (IQR = 8.6–37.7). External reliability of the Luganda and Kiswahili versions was assessed by calculating an Intra-Class Coefficient (ICC; Table 1) using a two-way mixed-effects model with absolute agreement for single measurements. For both Luganda and Kiswahili versions ICC was > 0.8. Kappa values for individual items are presented in supplementary Table S5. Values for the ‘reality’ and ‘congruence’ subscales indicated substantial response agreement, whilst fair to moderate agreement was observed for the ‘confront others’ subscale. These data support good consistency and external reliability of the translated scales. In places Kappa values may appear counterintuitive relative to observed agreement (e.g. “Pregnancy not seem real”, Kappa = 0 for Ugandan participants) due to prevalence and marginal distributions and should be interpreted cautiously.

**Table 1:** Test-retest reliability for translated PGIS questionnaires

Test-retest results	PGIS T1 Mean (SD)	PGIS T2 Mean (SD)	ICC (95% CI)	*p*-value
Kenya (*n* = 10)	3.41 (0.272)	3.51 (0.246)	0.819 (0.337, 0.954)	0.007
Uganda (*n* = 10)	3.49 (0.205)	3.41 (0.181)	0.908 (0.379, 0.980)	< 0.001
Overall (*n* = 20)	3.45 (0.238)	3.46 (0.216)	0.840 (0.590, 0.937)	< 0.001

PGIS results in pilot testing cohort. ICC was calculated using two-way mixed-effects model with absolute agreement for single measurements. P-value from F test of the null hypothesis that ICC = 0

### Wider Testing

#### Demographics characteristics

A total of 160 women participated in testing of the finalised tools (Table 2): 20 women recruited to pilot testing (where only baseline results from the test-retest were included in the overall results), and 140 recruited for wider testing (see Fig. 1). The median age was 29 (IQR = 24-34.5) and women had a median of 10 (IQR = 8–12) years of education. Most women were married (*n* = 128/160, 80%) and employed (*n* = 83/160, 52%). More babies were stillborn (*n* = 97/160, 61%) than died in the neonatal period (*n* = 63/160, 39%). The demographic characteristics of test-retest (*n* = 20) sample are detailed in supplementary table S4.

**Table 2:** Demographic characteristics, Testing phase

Characteristic	Overall *N* = 160	Kenya *N* = 80	Uganda *N* = 80
Age in years, median (IQR)	29 (24-34.5)	31 (26-36.5)	25 (22-31.5)
Years of schooling, median (IQR)	10 (8–12)	10.5 (8–12)	10 (7-12.5)
Weeks of gestation, median (IQR)	39.5 (30.5–40)	40 (32–40)	39 (29–40)
Time since loss (months), median (IQR)	7.1 (2.2–15.9)	10.8 (5.6–18.4)	2.3 (2.1–12.2)
Religion, n (%)			
Christian	143 (89%)	79 (99%)	64 (80%)
Muslim	17 (11%)	1 (1%)	16 (20%)
Marital status, n (%)			
Single	21 (13%)	15 (19%)	6 (8%)
Married/living with partner	128 (80%)	63 (79%)	65 (81%)
Widowed	1 (1%)	1 (1%)	0 (0%)
Separated /divorced	10 (6%)	1 (1%)	9 (11%)
Employment, n (%)			
Employed	83 (52%)	38 (47%)	45 (56%)
Unemployed	45 (28%)	35 (44%)	10 (13%)
Homemaker	32 (20%)	7 (9%)	25 (31%)
Occupation, n (%)			
Business	42 (51%)	14 (37%)	28 (62%)
Professional	14 (17%)	4 (11%)	10 (22%)
Other semi-skilled	10 (12%)	5 (13%)	5 (11%)
Labourer	15 (18%)	15 (39%)	0 (0%)
Subsidence farmer	2 (2%)	0 (0%)	2 (5%)
Type of death, n (%)			
Antenatal stillbirth	51 (32%)	27 (34%)	24 (30%)
Intrapartum stillbirth	46 (29%)	30 (38%)	16 (20%)
Neonatal death	63 (39%)	23 (29%)	40 (50%)
Sex of baby			
Male	91 (57%)	41 (51%)	50 (63%)
Female	68 (43%)	38 (48%)	30 (38%)
Type of birth			
Normal birth	124 (78%)	62 (77%)	62 (77%)
C-section	36 (22%)	18(23%)	18 (23%)

Table 2 shows the Demographics from pooled test-retest and wider-testing phase participants.

#### Internal consistency

The mean PGIS score was 3.43[0.268] (mean [SD] *n* = 160), scores in Uganda (*n* = 3.49 [0.229]), were higher than in Kenya (*n* = 3.37 [0.291]) (Table 3). PGIS scores showed no strong evidence of departure from normality (skewness–kurtosis test).

**Table 3:** Overall mean PGIS scores

	PGIS Scores - *n*, Mean (SD)			*p*-value
	Pooled	Pilot testing	Wider testing	
Kenya	*n* = 80; 3.37 (0.291)	*n* = 10; 3.41 (0.272)	*n* = 70; 3.36 (0.295)	0.607
Uganda	*n* = 80; 3.49 (0.229)	*n* = 10; 3.49 (0.205)	*n* = 70; 3.49 (0.234)	0.971
Overall	*n* = 160; 3.43 (0.268)	*n* = 20; 3.45 (0.238)	*n* = 140; 3.43 (0.273)	0.708

PGIS scores in pilot- and wider-testing participants, and pooled. P-value is t-test comparing mean PGIS in pilot- and wider-testing

Cronbach’s Alpha for the total PGIS scale was 0.737, with subscale alphas 0.657 (reality), 0.643 (confront others) and 0.685 (congruence), demonstrating acceptable internal consistency across the scale (Table 4). The magnitude of the average inter-item covariances suggests adequate homogeneity among items within each subscale. Similar results were observed for the pilot-testing and wider-testing subgroups.

**Table 4:** Cronbach’s Alpha for the total PGIS and Sub-scales scores

	Pooled		Pilot testing		Wider testing	
Subscales	Average inter-item co-variance	Cronbach’s Alpha	Average inter-item co-variance	Cronbach’s Alpha	Average inter-item co-variance	Cronbach’s Alpha
Reality	0.217	0.657	0.353	0.756	0.199	0.639
Confront others	0.380	0.643	0.258	0.581	0.419	0.666
Congruence	0.402	0.685	0.443	0.656	0.405	0.715
Total PGIS	0.177	0.737	0.210	0.763	0.175	0.740

Interitem covariance and Cronbach Alpha in the 20 pilot and 140 wider testing participants

**Fig. 2:**
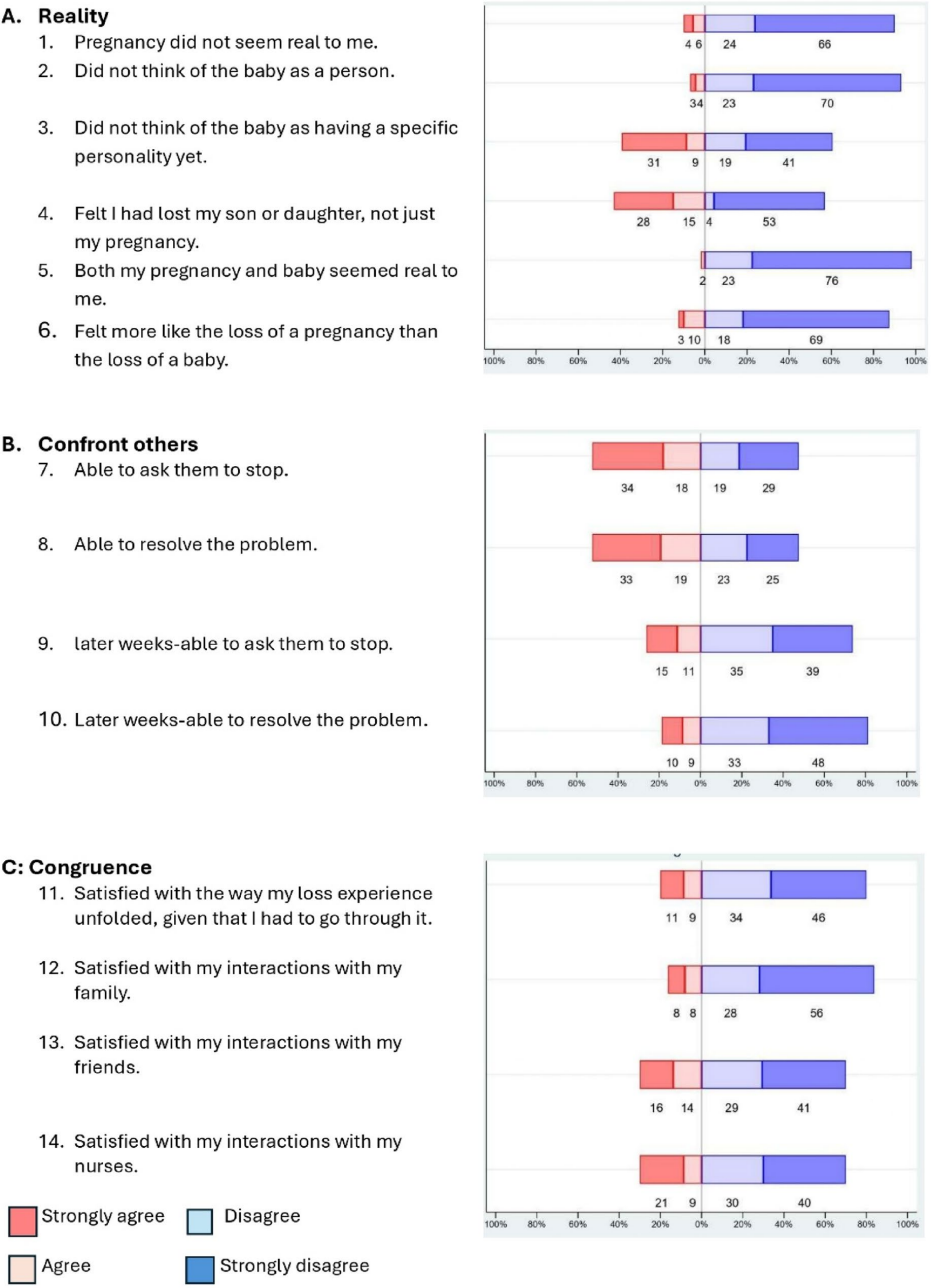
PGIS responses by subscale and item % of participants (N=160) from pooled pilot- and wider-testing participants, responding strongly agree, agree, disagree and strongly disagree

Figure 2 presents the spread and direction of responses for each subscale. Details of country specific findings for each sub scale are shown in supplementary figures S1 and S2. 

#### Single variable regression

The relationship between PGIS score and participant characteristics was explored using single variable linear regression, with the PGIS score as the dependent variable (Table 5). There was a suggestion that total PGIS score increased for every week of gestational age at birth/death of the baby (coefficient 0.008 (95%CI: 0.000 to 0.008, *p* = 0.054), and decreased for each previous pregnancy (coefficient − 0.026 (95%CI: -0.052 to 0.001, *p* = 0.058). Total PGIS score was higher for women in the Uganda site than in the Kenya site by 0.123 (95%CI: 0.042 to 0.205), *p* = 0.003] and for women birthing by caesarean Sect.  0.113 (95%CI: 0.014 to 0.211), *p* = 0.025] compared to vaginal birth.

**Table 5:** Single variable regression of PGIS score and Participant characteristics

Dependent variable: PGIS score			
Continuous variables		Coefficient (95% CI)	*P* value
Age (years)		-0.041 (-0.099, 0.017)	0.169
Years schooling		0.009 (-0.004, 0.021)	0.178
Weeks pregnant		0.008 (-0.000, 0.016)	0.054
Previous pregnancies		-0.026 (-0.052, 0.001)	0.058
Living children		-0.016 (-0.047, 0.016)	0.337
Time since loss (months)		-0.000 (-0.004, 0.003)	0.927

Categorical variables		**Coefficient (95% CI)**	**P value**
Research site	Kenya	-	0.003*
	Uganda	0.123 (0.042, 0.205)	
Religion	Christian	-	0.894
	Muslim	0.009 (-0.126, 0.145)	
Marital status	Single	-	0.872
	Living with partner	-0.096 (-0.341, 0.150)	
	Married	0.016 (-0.109, 0.142)	
	Widowed	-0.105 (-0.648, 0.438)	
	Separated/divorced	0.028 (-0.176, 0.232)	
Employment	Full time	-	0.216
	Part time	-0.108 (-0.232, 0.016)	
	Unemployed	-0.095 (-0.200, 0.009)	
	Homemaker	-0.061 (-0.177, 0.054)	
Type of death	Antepartum Stillbirth	-	0.449
	Intrapartum Stillbirth	0.015 (-0.092, 0.122)	
	Neonatal death	0.061 (-0.038, 0.160)	
Sex of baby	Male	-	0.431
	Female	-0.002 (-0.008, 0.003)	
Type of birth	Vaginal birth	-	0.025*
	Caesarean Section	0.113 (0.014, 0.211)	
Pregnancy complications	No	-	0.176
	Yes	0.076 (-0.034, 0.187)	
Previous SB or NND*	No	-	0.263
	Yes	-0.062 (-0.170, 0.046)	

Results from linear single-variable regression in n=160 pooled pilot- and wider-testing participants (pilot-testing uses first PGIS result only). p value from an omnibus F test for categorical variables

*NND Neonatal death, *SB Stillbirth

A sensitivity regression analysis was performed using results from the 140 women recruited for wider testing only (Supplementary Table S9). The effect of gestational age was less in this sub-group (coefficient 0.006, (95%CI: -0.003 to 0.015, *p* = 0.167); and for caesarean section (coefficient 0.088 (95%CI: -0.026 to 0.202, *p* = 0.131. Results for number of previous pregnancies and site were similar.

## Discussion

The aim of this study was to translate and conduct an initial psychometric evaluation of Kiswahili and Luganda versions of the PGIS scale. The translation process involving systematic, forward and back translations and expert/community member review generated agreed, context appropriate wording which required only minor adjustment in pilot testing. The translations were agreed by the panel of multidisciplinary stakeholders and CEI members which achieved semantic, idiomatic, experiential and conceptual equivalence to the source items [20]. Furthermore, the adjustments made ensured cultural sensitivity and accurately captured the experiences of women who experience perinatal deaths in the research settings contributing to face and content validity [24]. Preliminary psychometric evaluation with women who experienced stillbirth or newborn death demonstrated that the translated tools had good external reliability (ICC = 0.840 (0.590, 0.937) and acceptable internal consistency (Cronbach’s α = 0.737). These results suggest that the translated tools have potential as reliable measures of perinatal grief intensity in these contexts [15], .

From a feasibility perspective, the PGIS is brief and easy to score as compared to other instruments [11]. The lower response burden may favour the use of this scale for translation and adaptation in low-resource settings where response fatigue might be compounded by varying levels of literacy [25]. The constructs of the PGIS focus on how the death of the baby is perceived rather than the loss itself. Responses to perinatal bereavement are significantly impacted by social and cultural factors varying even within countries or regions, which could impact the transferability of instruments developed in different contexts. However, our previous qualitative research demonstrated a commonality in cultural influences on parents’ experiences in Kenya and Uganda [26]. Considerable resonance was also observed with studies across global settings with women’s experiences in Kenya and Uganda after the death of their baby [27]. Women in both countries described profound emotional distress, stigma and silence around baby death leading to disenfranchised grief [27, 28]. Women’s experiences also highlight the important influence of feelings about the pregnancy, availability and quality of support from health workers and responses of family, friends and wider social networks [27–29]. The findings resonate with the theoretical framework underpinning development of the PGIS and relevance to assessment of grief intensity in Kenya and Uganda settings.

Although the PGIS was initially developed for use after a miscarriage, it has been shown to be highly reliable and valid in measuring grief after other types of losses including stillbirths or neonatal deaths in the original high income country context [16]. The Cronbach’s alpha value for this study exceeds the threshold for acceptable internal consistency [30], in line with previous psychometric evaluation of English versions of the PGIS [15, 16, 31]. Evaluation of the PGIS in Mandarin and Spanish produced comparable internal consistency, with overall Cronbach’s alphas of 0.768 and 0.69 respectively [32, 33]. Despite the robust translation processes, cultural and conceptual nuances in the expression of grief; for example, lack of direct equivalent terms in Kiswahili and Luganda may have impacted the item-total correlations and the observed alpha values.

We observed higher overall mean PGIS scores for women in the site in Uganda, than in Kenya, the reasons for this are not clear. There were no notable differences in other maternal characteristics. Since participants were recruited from one site in each country, differences may not reflect country contexts but could have been confounded by other factors such as language, care pathways, services or sampling between the two sites. Women who had birthed by caesarean section had higher scores than women birthing vaginally. In LMICs, caesarean section often reflects traumatic birth experiences, particularly in the context of stillbirth or newborn death, and also increases physical and psychological challenges of recovery [34]. Independent of outcome, caesarean birth is associated with increased risk of postpartum depression and post-traumatic stress disorder with higher risks for unplanned caesarean birth [35, 36].

A study including 103 English-speaking women after miscarriage, stillbirth or newborn death described cut off values for PGIS indicating high grief intensity, at ≥ 3.535 [16]. Scores above this threshold were associated with subsequent development of severe depressive symptoms and intense anxiety at 3 to 5 months after the loss in a US sample [11]. The PGIS has been translated and validated with Chinese and Spanish women [32, 33], but no study has yet explored cut off values in different populations. Further studies would be needed to establish the predictive value of the Luganda and Kiswahili versions of PGIS in identifying clinically meaningful grief intensity and subsequent psychological distress in Kenya and Uganda.

### Strengths and weaknesses

This study followed a systematic approach to cross-cultural translation which was key to producing a high-quality translation. This was supported by inclusion of multiple translators with expertise in both source and target languages [37]. A particular strength was community and stakeholder involvement which is often neglected in psychometric evaluation studies [24]. The multidisciplinary expert panels comprised health workers, researchers and parents with experience of death of a baby, combining topic and language expertise. Community representatives, including those of different ethnic backgrounds, were able to advise on words or phrases which could not be directly translated or were unclear. This was important to ensure that translations could be understood by women whose first language was not Kiswahili or Luganda.

The sample size for the assessment of consistency was relatively small, leading to potential uncertainty in these values. Evaluation of the final versions of the tools involved women who had experienced a stillbirth or neonatal death over a range of time intervals, including recent losses and some more than a year previously. However, scores for participants in the test-retest phase (losses from 8 to 37 months ago) were not significantly different to those recruited in wider test phase, most of whom had experienced the death of the baby within 7 months prior to participation. The majority of the women were recruited from urban facilities, potentially limiting the diversity of experiences and transferability to those living in rural settings. While our study establishes initial reliability of the Luganda and Kiswahili versions of the PGIS among bereaved postnatal women, further work is still needed to confirm structural validity, convergent validity, and equivalence to provide further evidence for validity as a robust assessment of grief across settings.

## Conclusion

The Kiswahili and Luganda versions of the PGIS showed good reliability for use in Kenya and Uganda settings. The translated tools have potential for future use in clinical practice and research across similar contexts, to screen for intense grief among bereaved women and identify the need for follow-up and psychological support after a perinatal loss. Further research is required to establish structural validity, predictive performance, including cut off values and cross-language equivalence.

## Supplementary information

Supplementary Material 1.

## Data Availability

The datasets generated and/or analysed during the current study are not archived publicly but are available from the corresponding author on reasonable request.

## Abbreviations

PGS: Perinatal Grief Scale
PGIS: Perinatal Grief Intensity Scale
CEI: Community Engagement and Involvement
NIHR: National Institute for Health and Care Research
ICC: Intraclass Correlation Coefficient
