# Supplementary material for: Translation and evaluation of the Perinatal Grief Intensity Scale in Kenya and Uganda
Source: BMC Pregnancy Childbirth. 2026 Jun 8;26:909. doi: 10.1186/s12884-026-09394-6 (PMC13483843; doi:10.1186/s12884-026-09394-6)
Supplement: Supplementary file 1 [file 12884_2026_9394_MOESM1_ESM.docx]

**Supplementary Material**

**Table S1:** Adjustments made to the Kiswahili translation following the expert panel review.

| **Original English terms** | **Initial translation** | **Changes made** | **Consensus translation** |
| --- | --- | --- | --- |
| **Item 1:** The pregnancy did not seem real to me. | Ujauzito huo hukuonekana kuwa wa kweli | The word “Nilifikiria” (a translation for ‘I thought’) was added at the beginning. | Nilifikiria ujauzito huo hukuonekana kuwa wa kweli |
| **Item 5:** Both my pregnancy and baby seemed real to me. | Uja uzito wangu na mtoto wangu walionekana kuwa halisi kwangu. | Accepted as it was but rephrased ‘walionekana’ to ‘ulionekana’ (a translation for ‘it appeared’). | Uja uzito wangu na mtoto wangu ulionekana kuwa halisi kwangu. |
| **Item 6:** It seemed more like the loss of a pregnancy than the loss of a baby. | Ilionekana zaidi kama kupoteza uja uzito kuliko mtoto kuaga. | Left as it was but removed the word *“kuaga”* which refers to death. This word was thought to be stigmatizing for women who were already stigmatized from the facility and their homes. | Ilionekana zaidi kama kupoteza uja uzito kuliko mtoto. |
| **Item 7:** In the first hours and days after my loss, if people said or did things that made me feel bad, I was able to ask them to stop. | Katika masaa ya kwanza na siku za kwanza baada ya kupoteza mimba au mtoto kuaga, ikiwa watu walisema au kufanya mambo ambayo yalinifanya nihisi vibaya, niliweza kuwaulia waache. | Left as it was but removed the word “kuaga”. | Katika masaa ya kwanza na siku za kwanza baada ya kupoteza mimba au mtoto, ikiwa watu walisema au kufanya mambo ambayo yalinifanya nihisi vibaya, niliweza kuwaulia waache. |
| **Item 8:** In the first hours and days after my loss, if something happened that I did not like, I was usually able to resolve the problem. | Katika masaa ya kwanza na siku za kwanza baada ya kupoteza mimba au mtoto kuaga, ikiwa kitu kilitendeka ambacho sikukipenda, nilikuwa kwa kawaida niliweza kutatua tatizo hilo. | Left as it was but removed the word “kuaga”. | Katika masaa ya kwanza na siku za kwanza baada ya kupoteza mimba au mtotoa, ikiwa kitu kilitendeka ambacho sikukipenda, nilikuwa kwa kawaida niliweza kutatua tatizo hilo. |
| **Item 9:** In later weeks after my loss, if people said or did things that made me feel bad, I was able to ask them to stop. | Katika wiki za baadaye baada ya kupoteza mimba au mtoto kuaga ikiwa watu walisema au kufanya mambo ambayo yalinifanya nijiskie vibaya, niliweza kuwauliza waache. | Left as it was but removed the word “kuaga”. | Katika wiki za baadaye baada ya kupoteza mimba au mtoto ikiwa watu walisema au kufanya mambo ambayo yalinifanya nijiskie vibaya, niliweza kuwauliza waache. |
| **Item 10:** In later weeks after my loss, if something happened that I did not like, I was usually able to resolve the problem. | Katika wiki za baadaye baada ya kupoteza mimba au mtoto kuaga ikiwa kitu kingefanyika ambacho sikukipenda, kwa kawaida niliweza kutatua tatizo hilo. | Left as it was but removed the word “kuaga”. | Katika wiki za baadaye baada ya kupoteza mimba au mtoto ikiwa kitu kingefanyika ambacho sikukipenda, kwa kawaida niliweza kutatua tatizo hilo. |
| **Item 11:** During and after my perinatal loss, I was satisfied with the way my loss experience unfolded, given that I had to go through it. | Punde tu na baada ya kupoteza mimba au mtoto wangu kuaga, niliridhika kwa hayo yaliyotendeka nikikubali kwamba nilipaswa kuyapitia. | Left as it was but removed the word “kuaga”. | Punde tu na baada ya kupoteza mimba au mtoto wangu, niliridhika kwa hayo yaliyotendeka nikikubali kwamba nilipaswa kuyapitia. |
| **Item 12:** During and after my perinatal loss, I was satisfied with my interactions with my family. | Punde tu na baada ya kupoteza mimba au mtoto wangu kuaga, niliridhika na utangamano wangu na familia yangu. | Left as it was but removed the word “kuaga”. | Punde tu na baada ya kupoteza mimba au mtoto wangu, niliridhika na utangamano wangu na familia yangu. |
| **Item 13:** During and after my perinatal loss, I was satisfied with my interactions with my friends. | Punde tu na baada ya kupoteza mimba au mtoto wangu kuaga niliridhika na utangamano wangu na marafiki zangu. | Left as it was but removed the word “kuaga”. | Punde tu na baada ya kupoteza mimba au mtoto wangu niliridhika na utangamano wangu na marafiki zangu. |
| **Item 14:** During and after my perinatal loss, I was satisfied with my interactions with my nurses. | Punde tu na baada ya kutoteza mimba au mtoto wangu kuaga, niliridhika na utangamano wangu na wauguzi. | Left as it was but removed the word “kuaga”. | Punde tu na baada ya kutoteza mimba au mtoto wangu, niliridhika na utangamano wangu na wauguzi. |

**Table S2:** Adjustments made to the Luganda translation following the expert panel review.

| **Original English terms** | **Initial translation** | **Changes made** | **Consensus translation** |
| --- | --- | --- | --- |
| Perinatal Grief Intensity Scale | Minzaani ekozesebwa okupima obungi bw’okunyolwa oluvanyuma lw’okufiirwa omwana. | This was changed to “Ebibuuzo ku kunyolwa eri abo abafiiriddwa abaana” in order to fit in the context of a loss. | Ebibuuzo ku kunyolwa eri abo abafiiriddwa abaana. |
| **Item 1:** The pregnancy did not seem real to me. | Olubuto telwandabikira kuba nga lwa ddala. | Edited and replaced “telwandabikira” (a translation for ‘didn’t appear to be’) with “lwali nga” (a translation for “it was as though”) which would be easy for the women to understand. | Olubuto lwali nga olutali lwa ddala. |
| **Item 2:** I did not think of the baby as a person. | Sakilowooza nti omwana yali muntu wa ddala. | Accepted as it was but added “afuuse” and removed “wa ddala”. | Sakilowooza nti omwana yali afuuse omuntu. |
| **Item 3:** I did not think of the baby as having a specific personality yet. | Omwana eyali mu lubuto nnali simusubira kw’eyisa ng’omuntu. | Accepted as it was but replaced “ssimusuubira kw’eyisa ng’omuntu” with “ssinnaba kumulowozaako ng’omuntu”. | Omwana eyali mu lubuto nali ssinnaba kumulowozaako ng’omuntu. |
| **Item 4:** I felt I had lost my son or daughter, not just my pregnancy. | Nnawulira nga aviiriddwaako mutabani oba muwala wange, wabula ssi lubuto lwange lwokka. | Replaced “aviiriddwaako” with “afiiriddwa” and “lubuto lwange lwokka” with “Lubuto bu buto” | Nnawulira nga afiiriddwa mutabani/muwala wange wabula ssi lubuto bu buto. |
| **Item 5:** Both my pregnancy and baby seemed real to me. | Olubuto lwange wamu n’omwana nali mpulira nga byonna bya ddala. | Accepted as it was, but replaced “byonna” (a translation for ‘all’) with “byombi” (a translation for ‘both)’. | Olubuto lwange wamu n’omwana nali mpulira nga byombi bya ddala. |
| **Item 6:** It seemed more like the loss of a pregnancy than the loss of a baby. | Kyandabikira nnyo nga eyavaamu olubuto wabula ssi nga eyafiirwa omwana. | Accepted as it was, but replaced “kyandabikira nnyo” with “nawulira nga”. | Nawulira nga eyavaamu olubuto sso ssi nga eyafiirwa omwana. |
| **Item 7:** In the first hours and days after my loss, if people said or did things that made me feel bad, I was able to ask them to stop. | Mu ssawa n’ennaku ez’asooka nga n’akafiirwa, abantu bweba’yogera nga oba n’ebakola ebintu ebyandetera okuwulira obubi, nasobola nga okubasaba bakikomye. | Accepted as it was, but replaced “mu ssaawa” with “mu kiseera” | Mu kiseera n’ennaku ezasooka nga n’akafiirwa, abantu bweba’yogera nga oba n’ebakola ebintu ebyandetera okuwulira obubi, nasobola nga okubasaba bakikomye. |
| **Item 8:** In the first hours and days after my loss, if something happened that I did not like, I was usually able to resolve the problem. | Mu ssawa n’ennaku ez’asooka nga n’akafiirwa, ekintu ky’essaagala bweky’aberanga wo, nasobola nga okukigonjoola. | Accepted as it was, but replaced “mu ssaawa” with “mu kiseera” | Mu kiseera n’ennaku ezasooka nga n’akafiirwa, ekintu ky’essaagala bweky’aberanga wo, nasobola nga okukigonjoola. |
| **Item 9:** In later weeks after my loss, if people said or did things that made me feel bad, I was able to ask them to stop. | Mu sabbiiti ezaddilira nga n’akafiirwa, abantu bweba’yogera nga oba n’ebakola ebintu ebyandetera okuwulira obubi, nasobola nga okubasaba bakikomye. | Accepted as it was but replaced “sabbiiti” with “wiiki”. | Mu wiiki ezaddirira nga n’akafiirwa, abantu bwe baayogera nga oba n’ebakola ebintu ebyandetera okuwulira obubi, nasobola nga okubasaba bakikomye. |
| **Item 10:** In later weeks after my loss, if something happened that I did not like, I was usually able to resolve the problem. | Mu sabbiiti ezaddilira nga n’akafiirwa, ekintu ky’essaagala bweky’aberanga wo, nasobola nga okukigonjoola. | Accepted as it was, but replaced “sabbiiti” with “wiiki”. | Mu wiiki ezaddilira nga n’akafiirwa, ekintu ky’essaagala bweky’aberanga wo, nasobola nga okukigonjoola. |
| **Item 11:** During and after my perinatal loss, I was satisfied with the way my loss experience unfolded, given that I had to go through it. | Mukiseera n’oluvannyuma lw’okufiirwa omwana, nnali mumativu n’engeri byennayitamu gye byabaawo, olw’okuba nti nnali nnina okubiyitamu. | Accepted as it was | Mu kiseera ekyo n’oluvannyuma lw’okufiirwa omwana, nnali mumativu n’engeri byennayitamu gye babaawo, olw’okuba nti nnali nnina okubiyitamu. |
| **Item 12:** During and after my perinatal loss, I was satisfied with my interactions with my family. | Mukiseera n’oluvanyuma lw’okufiirwa omwana, nnali mumativu n’enkolagana yange n’abomumaka gange. | Accepted as it was, but added the word “okumbererawo” [which means “support”] in the Luganda translation so that it makes more sense to the Ugandan women. | Mu kiseera ekyo n’oluvanyuma lw’okufiirwa omwana, nnali mumativu n’enkolagana wamu n’engeri ab’omumaka gange gyebambererawo. |
| **Item 13:** During and after my perinatal loss, I was satisfied with my interactions with my friends. | Mukiseera n’oluvanyuma lw’okufiirwa omwana, nnali mumativu n’enkolagana yange ne mikwano gyange. | Accepted as it was but added the word “okumbererawo” [which means “support”] in the Luganda translation so that it makes more sense to the Ugandan women. | Mu kiseera ekyo n’oluvanyuma lw’okufiirwa omwana, nnali mumativu n’enkolagana wamu n’engeri mikwano gyange gye gyambererawo. |
| **Item 14:** During and after my perinatal loss, I was satisfied with my interactions with my nurses. | Mukiseera n’oluvannyuma lw’okufiirwa omwana, nnali mumativu n’enkolagana yange n’abasawo. | Accepted as it was. | Mukiseera n’oluvannyuma lw’okufiirwa omwana, nnali mumativu n’enkolagana yange n’abasawo. |

**Table S3:** Modifications made in the Luganda version of the PGIS following feedback from the Think Aloud Interviews.

| **Item/ Words** | **Initial translation** | **Comment** | **Final Translation** |
| --- | --- | --- | --- |
| **Title of the tool:** Perinatal Grief Intensity Scale | Ebibuuzo ku kunyolwa eri abo abafiiriddwa abaana | Needed to add that the loss was either before birth or shortly after birth. | Ebibuuzo ku kunyolwa eri abo abafiiriddwa abaana nga tebannazaalibwa oba mu kiseera kitono nga bakazaalibwa. |
| **Item 3:** I did not think of the baby as having a specific personality yet. | Omwana eyali mulubuto nali ssinnaba kumulowozaako ng’omuntu | Some women felt like item 2 and 3 were similar. The research team revisited the translation for item 3 and modified it to ensure that it was capturing its intended meaning | Sakilowooza nti omwana yali atandise okuba n’embeera ze ng’omuntu. |
| **Item 6:** | Nawulira nga eyavaamu olubuto sso ssi nga eyafiirwa omwana. | Needed to change the tense in the initial translation of ‘lost’ | Nawulira nga eyavaamu olubuto sso ssi nga afiiriddwa omwana. |
| **Solve** | Okukigonjoola | This term was reported to be difficult for some women. However, the research team did not change it since there was no alternative to it in the local language. | |

**Table S4:** Demographic characteristics of participants in the Test-retest phase

|  |  | Overall N=20 | Kenya N=10 | Uganda N=10 |
| --- | --- | --- | --- | --- |
| Age, median (IQR) | | 31 (26.5-36) | 34.5 (31-40) | 27 (24-31) |
| Years schooling, median (IQR) | | 11 (8-12) | 12 (8-12) | 11 (7-11) |
| Weeks pregnant, median (IQR) | | 40 (31-40) | 35 (30-41) | 40 (40-40) |
| Time since loss (months), median (IQR) | | 25.4 (8.6-37.7) | 12.2 (6.6-47.8) | 26.0 (25.2-32.8) |
|  |  |  |  |  |
| Religion, n (%) | |  |  |  |
|  | Christian | 19 (95%) | 10 (100%) | 9 (90%) |
|  | Muslim | 1 (5%) | 0 (0%) | 1 (10%) |
| Marital status, n (%) | |  |  |  |
|  | Single | 4 (20%) | 3 (30%) | 1 (10%) |
|  | Living with partner | 0 (0%) | 0 (0%) | 0 (0%) |
|  | Married | 15 (75%) | 7 (70%) | 8 (80%) |
|  | Widowed | 0 (0%) | 0 (0%) | 0 (0%) |
|  | Separated/divorced | 1 (5%) | 0 (0%) | 1 (10%) |
| Employment, n (%) | |  |  |  |
| Employed | | 10 (50%) | 4 (40%) | 6 (60%) |
|  | Unemployed | 3 (15%) | 2 (20%) | 1 (10%) |
|  | Homemaker | 7 (35%) | 4 (40%) | 3 (30%) |
| Occupation, n (%) | |  |  |  |
|  | Business | 6 (60%) | 2 (50%) | 4 (67%) |
|  | Labourer | 1 (10%) | 1 (25%) | 0 (0%) |
|  | Laundry | 1 (10%) | 1 (25%) | 0 (0%) |
|  | Maintenance officer | 1 (10%) | 0 (0%) | 1 (17%) |
|  | Secretarial | 1 (10%) | 0 (0%) | 1 (17%) |
| Type of death, n (%) | |  |  |  |
|  | Antenatal stillbirth | 7 (35%) | 5 (50%) | 2 (20%) |
|  | Intrapartum stillbirth | 7 (35%) | 3 (30%) | 4 (40%) |
|  | Neonatal death | 6 (30%) | 2 (20%) | 4 (40%) |
| Sex | |  |  |  |
|  | Male | 12 (60%) | 6 (60%) | 6 (60%) |
|  | Female | 8 (40%) | 4 (40%) | 4 (40%) |
| Type of birth | |  |  |  |
|  | Normal birth | 11 (55%) | 6 (60%) | 5 (50%) |
|  | C-section | 9 (45%) | 4 (40%) | 5 (50%) |

**Table S5:** Kappa values for individual components

| **Subscale/Item** | **Kenya (n=10)** | **Uganda (n=10)** | **Overall (n=20)** |
| --- | --- | --- | --- |
| **Reality** | | | |
| 1. Pregnancy did not seem real | Agree=90% Kappa=0.737 | Agree=90% Kappa=0.000 | Agree=90% Kappa=0.608 |
| 2. Don't think of baby as person | Agree=90% Kappa=0.677 | Agree=100% Kappa=1.000 | Agree=95% Kappa=0.762 |
| 3. Don't think baby has specific personality | Agree=80% Kappa=0.333 | Agree=90% Kappa=0.600 | Agree=90% Kappa=0.795 |
| 4. Felt I had lost my son or daughter | Agree=80% Kappa=0.000 | Agree=100% Kappa=1.000 | Agree=95% Kappa=0.900 |
| 5. Both pregnancy and baby seemed real | Agree=80% Kappa=0.375 | Agree=100% Kappa=1.000 | Agree=90% Kappa=0.608 |
| 6. More like loss of pregnancy than of baby | Agree=80% Kappa=0.545 | Agree=100% Kappa=1.000 | Agree=90% Kappa=0.677 |
| **Confront others** | | | |
| 7. Able to ask others to stop | Agree=67% Kappa=0.265 | Agree=70% Kappa=0.375 | Agree=68% Kappa=0.275 |
| 8. Able to resolve problems | Agree=80% Kappa=0.423 | Agree=70% Kappa=0.286 | Agree=75% Kappa=0.345 |
| 9. Later weeks - able to ask others to stop | Agree=87% Kappa=0.487 | Agree=80% Kappa=0.524 | Agree=90% Kappa=0.565 |
| 10. Later weeks - able to resolve problems | Agree=87% Kappa=0.655 | Agree=75% Kappa=0.167 | Agree=85% Kappa=0.516 |
| **Congruence** | | | |
| 11. Satisfied with how loss experience unfolded | Agree=97% Kappa=0.923 | Agree=95% Kappa=0.844 | Agree=97% Kappa=0.923 |
| 12. Satisfied with interactions with family | Agree=93% Kappa=0.756 | Agree=95% Kappa=0.773 | Agree=95% Kappa=0.840 |
| 13. Satisfied with interactions with friends | Agree=80% Kappa=0.531 | Agree=90% Kappa=0.783 | Agree=87% Kappa=0.698 |
| 14. Satisfied with interactions with nurses | Agree=87% Kappa=0.677 | Agree=93% Kappa=0.848 | Agree=90% Kappa=0.777 |

**Table S6: Final Luganda version of the Perinatal Grief Intensity Scale**

**Ebibuuzo ku kunyolwa eri abo abafiiriddwa abaana** **nga tebannazaalibwa oba mu kiseera kitono nga bakazaalibwa.**

**Okunnyonnyola:**

**Okufiirwa omwana nga akyaali mu lubuto oba mu kiseera kitono nga yakazaalibwa kulimu ebintu bino wammanga:**

1. **Okuvaamu olubuto mu myezi egisooka** okugeza nga; **olubuto okuvaamu nga lukyaali luto oba olubuto okukulira mu nseke (olubuto oluvuddemu nga wiiki 28 tezinnaba kuwera) ne.**
2. **Okufiirwa omwana ng’olubuto nga lukuze oba mu bbanga ttono nga y’akazaalibwa.** Okugeza nga; **omwana okufiira mu lubuto** (omwana okufa nga tannazaalibwa naye nga wiiki 28 ziyiseeko) oba **omwana okufa mu kiseera kitono nga y’akazaalibwa** (gamba nga omwana okufa nga muwere - omwana okuzaalibwa nga mulamu naye n’afa mu nnaku 28 ezisooka).

***Ebibuuzo bino biyamba abasawo okuzuula obulungi abazadde abayiinza okuba n’okunyolwa okungi ennyo oluvannyuma lw’okufiirwa omwana, ng’olubuto lukyaali luto, oba nga lukuze, oba omwana okufa nga azaaliddwa.***

| **Endagiriro: Okuva ku by’okuddamu “Ssikiriziganyiza ddala nakyo” okutuuka ku “Nzikiriganyiza ddala nakyo”, teeka enkulungo ku ky’okuddamu ekiraga engeri gy’obadde owuliramu ku ky’okufiirwa omwana.** | | | | | | |
| --- | --- | --- | --- | --- | --- | --- |
| **I. Ekitundu ekisooka: Amazima ag’ebyaaliwo** | | | | | | |
| 1. Olubuto lwali nga olutali lwa ddala. | Ssikkiriziganyiza ddala nakyo  4 | Ssikkiriziganya nakyo  3 | Nzikiriziganya nakyo  2 | | Nzikiriziganyiza ddala nakyo  1 | |
| 2. Sakilowooza nti omwana yali afuuse omuntu. | Ssikkiriziganyiza ddala nakyo  4 | Ssikkiriziganya nakyo  3 | Nzikiriziganya nakyo  2 | | Nzikiriziganyiza ddala nakyo  1 | |
| 3. Sakilowooza nti omwana yali atandise okuba n’embeera ze ng’omuntu. | Ssikkiriziganyiza ddala nakyo  4 | Ssikkiriziganya nakyo  3 | Nzikiriziganya nakyo  2 | | Nzikiriziganyiza ddala nakyo  1 | |
| 4. Nnawulira nga afiiriddwa mutabani oba muwala wange, wabula ssi lubuto bu buto. | Ssikkiriziganyiza ddala nakyo  1 | Ssikkiriziganya nakyo  2 | Nzikiriziganya nakyo  3 | Nzikiriziganyiza ddala nakyo  4 | |  |
| 5. Olubuto lwange wamu n’omwana nali mpulira nga byombi bya ddala. | Ssikkiriziganyiza ddala nakyo  1 | Ssikkiriziganya nakyo  2 | Nzikiriziganya nakyo  3 | | Nzikiriziganyiza ddala nakyo  4 | |
| 6. Nawulira nga avuddemu olubuto naye ssi nga afiiriddwa omwana. | Ssikkiriziganyiza ddala nakyo  4 | Ssikkiriziganya nakyo  3 | Nzikiriziganya nakyo  2 | | Nzikiriziganyiza ddala nakyo  1 | |

**Omugatte ogusooka =	_______**

| **II. Ekitundu eky’okubiri: Okuyimirira n’obuvumu** | | | | |
| --- | --- | --- | --- | --- |
| 7**.** Mu kiseera n’ennaku ezasooka nga n’akafiirwa, abantu bwebayogera nga oba nebakola ebintu ebyandetera okuwulira obubi, nasobola nga okubasaba bakikomye. | Ssikkiriziganyiza ddala nakyo  4 | Ssikkiriziganya nakyo  3 | Nzikiriziganya nakyo  2 | Nzikiriziganyiza ddala nakyo  1 |
| 8. Mu kiseera n’ennaku ezasooka nga n’akafiirwa, ekintu kye ssaagala bwekyaberanga wo, nasobola nga okukigonjoola. | Ssikkiriziganyiza ddala nakyo  4 | Ssikkiriziganya nakyo  3 | Nzikiriziganya nakyo  2 | Nzikiriziganyiza ddala nakyo  1 |
| 9. Mu wiiki ezaddirira nga n’akafiirwa, abantu bwe baayogera nga oba nebakola ebintu ebyandetera okuwulira obubi, nasobola nga okubasaba bakikomye. | Ssikkiriziganyiza ddala nakyo  4 | Ssikkiriziganya nakyo  3 | Nzikiriziganya nakyo  2 | Nzikiriziganyiza ddala nakyo  1 |
| 10. Mu wiiki ezaddirira nga n’akafiirwa, ekintu kye ssaagala bwekyaberanga wo, nasobola nga okukigonjoola. | Ssikkiriziganyiza ddala nakyo  4 | Ssikkiriziganya nakyo  3 | Nzikiriziganya nakyo  2 | Nzikiriziganyiza ddala nakyo  1 |

**Omugatte ogusooka =	_______**

| **III. Ekitundu eky’okusatu: Obumativu n’engeri gyebambererawo.** | | | | |
| --- | --- | --- | --- | --- |
| 11. Mu kiseera ekyo n’oluvannyuma lw’okufiirwa omwana, nnali mumativu n’engeri byennayitamu gye byajja nga bibaawo, olw’okuba nti nnali nnina okubiyitamu. | Ssikkiriziganyiza ddala nakyo  4 | Ssikkiriziganya nakyo  3 | Nzikiriziganya nakyo  2 | Nzikiriziganyiza ddala nakyo  1 |
| 12. Mu kiseera ekyo n’oluvanyuma lw’okufiirwa omwana, nnali mumativu n’enkolagana wamu n’engeri ab’omumaka gange gye bambererawo. | Ssikkiriziganyiza ddala nakyo  4 | Ssikkiriziganya nakyo  3 | Nzikiriziganya nakyo  2 | Nzikiriziganyiza ddala nakyo  1 |
| 13. Mu kiseera ekyo n’oluvanyuma lw’okufiirwa omwana, nnali mumativu n’enkolagana wamu n’engeri mikwano gyange gye gyambererawo. | Ssikkiriziganyiza ddala nakyo  4 | Ssikkiriziganya nakyo  3 | Nzikiriziganya nakyo  2 | Nzikiriziganyiza ddala nakyo  1 |
| 14. Mu kiseera ekyo n’oluvannyuma lw’okufiirwa omwana, nnali mumativu n’enkolagana yange n’abasawo. | Ssikkiriziganyiza ddala nakyo  4 | Ssikkiriziganya nakyo  3 | Nzikiriziganya nakyo  2 | Nzikiriziganyiza ddala nakyo  1 |

**Omugatte ogusooka =	_______**

**Table S7: Final Kiswahili version of the Perinatal Grief Intensity Scale**

**Mizani ya kiwango cha ukali wa huzuni kabla, muda tu na baada ya kujifungua mtoto aliyeaga.**

**Ufafanuzi:**

Kupoteza mimba inahusu;

1. **Kupoteza mimba mapema** hii ni **kama Vile kuavya mimba** au **mimba nje ya nyungu ya uzazi** (mimba iliyotoka kabla ya wiki 28 za uja uzito), na
2. **Kupoteza mimba baadaye wa uja uzito au kuaga kwa mtoto baada tu ya kuzaliwa** (hii ni Vile kuaga kwa mtoto baada ya wiki 28 za uja uzito lakini kabla ya kuzaliwa kwake), **au kuaga kwa mtoto pindi tu unapojifungua** (ni kama Vile kifo cha watoto wachanga - kifo cha mtoto aliyekuwa hai siku za kwanza 28 baada ya kuzaliwa).

***Mizani hii huwasaidia madaktari na wauguzi kutambua vyema wazazi ambao wana uwezekano wa kuwa na makali ya huzuni baada ya kupoteza mimba mapema au kupoteza mimba badaye au kuaga kwa mtoto mchanga.***

| **MAELEKEZO:** Kwa mizani kutoka kwa "kutokubali kabisa" hadi "kukubali kabisa," weka mduara kuzunguka jibu ambalo liko karibu zaidi na jinsi umehisi kuhusu kupoteza mimba mapema au kupoteza mimba baadaye au kuaga kwa mtoto mchanga kabla ya kuishi zaidi ya siku 28. | | | | | | |
| --- | --- | --- | --- | --- | --- | --- |
| 1. **Mizani inayopima kiwango cha ukweli wa hisia kwa uwazi.** | | | | | | |
| 1. Ujauzito huo hukuonekana kuwa wa kweli. | Sikubaliani kabisa  4 | Sikubali  3 | Nakubali  2 | | Nakubali  Kabisa  1 | |
| 2. Sikumfikira mtoto huyo kama binadamu. | Sikubaliani kabisa  4 | Sikubali  3 | Nakubali  2 | | Nakubali  Kabisa  1 | |
| 3. Sikumfikiria mtoto bado kuwa na utu maalum. | Sikubaliani kabisa  4 | Sikubali  3 | Nakubali  2 | | Nakubali Kabisa  1 | |
| 4. Nilihisi kuwa nimempoteza mwana wangu wa kiume au binti yangu Sio uja uzito pekee. | Sikubaliani kabisa  1 | Sikubali  2 | Nakubali  3 | Nakubali Kabisa  4 | |  |
| 5. Uja uzito wangu na mtoto wangu walionekana kuwa halisi kwangu. | Sikubaliani kabisa  1 | Sikubali  2 | Nakubali  3 | | Nakubali  Kabisa  4 | |
| 6. Ilionekana zaidi kama kupoteza uja uzito kuliko mtoto kuaga. | Sikubaliani kabisa  4 | Sikubali  3 | Nakubali  2 | | Nakubali  Kabisa  1 | |

**JUMLA=**

| **II. Mizani inayopima kiwango cha kukubaliana na wengine.** | | | | |
| --- | --- | --- | --- | --- |
| 7**.** Katika masaa ya kwanza za kwanza na siku za kwanza baada ya kupoteza mimba au mtoto kaaga, ikiwa watu walisema au kufanya mambo ambayo yalinifanya nihisi vibaya, niliweza kuwaambia waache. | Sikubaliani kabisa  4 | Sikubali  3 | Nakubali  2 | Nakubali  Kabisa  1 |
| 8. Katika masaa ya kwanza na siku za kwanza baada ya kupoteza mimba au mtoto kaaga, ikiwa kitu kilitendeka ambacho sikukipenda, nilikuwa kwa kawaida niliweza kutatua tatizo hilo. | Sikubaliani kabisa  4 | Sikubali  3 | Nakubali  2 | Nakubali  Kabisa  1 |
| 9. Katika wiki za baadaye baada ya kupoteza mimba au mtoto kaaga ikiwa watu walisema au kufanya mambo ambayo yalinifanya nijiskie vibaya, niliweza kuwaambia waache. | Sikubaliani kabisa  4 | Sikubali  3 | Nakubali  2 | Nakubali  Kabisa  1 |
| 10. Katika wiki za baadaye baada ya kupoteza mimba au mtoto kaaga ikiwa kitu kingefanyika ambacho sikukipenda, kwa kawaida niliweza kutatua tatizo hilo | Sikubaliani kabisa  4 | Sikubali  3 | Nakubali  2 | Nakubali  Kabisa  1 |
| **JUMLA=** | | | | |
| III. **Mizani inayopima kiwango cha maelewanaa, ulinganifu na utangamano** | | | | |
| 11. Punde tu na baada ya kupoteza mimba au mtoto wangu kuaga, niliridhika na usaidizi niliopata. | Sikubaliani kabisa  4 | Sikubali  3 | Nakubali  2 | Nakubali  Kabisa  1 |
| 12.Punde tu na baada ya kupoteza mimba au mtoto wangu kuaga, niliridhika na ushirikiano wangu na familia yangu. | Sikubaliani kabisa  4 | Sikubali  3 | Nakubali  2 | Nakubali  Kabisa  1 |
| 13.Punde tu na baada ya kupoteza mimba au mtoto wangu kuaga niliridhika na ushirikiano wangu na marafiki wangu. | Sikubaliani kabisa  4 | Sikubali  3 | Nakubali  2 | Nakubali  Kabisa  1 |
| 14. Punde tu na baada ya kupoteza mimba au mtoto wangu kuaga, niliridhika na ushirikiano wangu na wauguzi. | Sikubaliani kabisa  4 | Sikubali  3 | Nakubali  2 | Nakubali  Kabisa  1 |

**JUMLA=**

**Table S8: Original English Version of the Perinatal Grief Intensity Scale**

**Perinatal Grief Intensity Scale**

**DEFINITIONS:**

**Perinatal losses include:**

1. **Early pregnancy losses** such as **miscarriage** or **ectopic pregnancy** (pregnancy loss before 28 weeks gestation), and
2. **Later losses in pregnancy or soon after birth** such as **stillbirth** (death of a baby after 28 weeks gestation but before birth), **or death of a baby soon after birth** (such as newborn death-death of a live-born baby in the first 28 days after birth).

***This scale helps doctors and nurses better identify parents who are likely to have intense grief after an early or late pregnancy loss or death of an infant.***

| **DIRECTIONS: On a scale from “strongly disagree” to “strongly agree,” place a circle around the answer that is closest to how you have felt about your perinatal loss.** | | | | |
| --- | --- | --- | --- | --- |
| **I. Reality Subscale** | | | | |
| 1. The pregnancy did not seem real to me. | Strongly Disagree  4 | Disagree  3 | Agree  2 | Strongly Agree  1 |
| 2. I did not think of the baby as a person. | Strongly Disagree  4 | Disagree  3 | Agree  2 | Strongly Agree  1 |
| 3. I did not think of the baby as having a specific personality yet. | Strongly Disagree  4 | Disagree  3 | Agree  2 | Strongly Agree  1 |
| 4. I felt I had lost my son or daughter, not just my pregnancy. | Strongly Disagree  1 | Disagree  2 | Agree  3 | Strongly Agree  4 |
| 5. Both my pregnancy and baby seemed real to me. | Strongly Disagree  1 | Disagree  2 | Agree  3 | Strongly Agree  4 |
| 6. It seemed more like the loss of a pregnancy than the loss of a baby. | Strongly Disagree  4 | Disagree  3 | Agree  2 | Strongly Agree  1 |

**Subtotal =	_______**

| **II. Confront Others Subscale** | | | | |
| --- | --- | --- | --- | --- |
| 7**.** In the first hours and days after my loss, if people said or did things that made me feel bad, I was able to ask them to stop. | Strongly Disagree  4 | Disagree  3 | Agree  2 | Strongly Agree  1 |
| 8. In the first hours and days after my loss, if something happened that I did not like, I was usually able to resolve the problem. | Strongly Disagree  4 | Disagree  3 | Agree  2 | Strongly Agree  1 |
| 9. In later weeks after my loss, if people said or did things that made me feel bad, I was able to ask them to stop. | Strongly Disagree  4 | Disagree  3 | Agree  2 | Strongly Agree  1 |
| 10. In later weeks after my loss, if something happened that I did not like, I was usually able to resolve the problem. | Strongly Disagree  4 | Disagree  3 | Agree  2 | Strongly Agree  1 |
| **Subtotal =** | | | | |
| **III. Congruence Subscale** |  |  |  |  |
| 11. During and after my perinatal loss, I was satisfied with the way my loss experience unfolded, given that I had to go through it. | Strongly Disagree  4 | Disagree  3 | Agree  2 | Strongly Agree  1 |
| 12. During and after my perinatal loss, I was satisfied with my interactions with my family. | Strongly Disagree  4 | Disagree  3 | Agree  2 | Strongly Agree  1 |
| 13. During and after my perinatal loss, I was satisfied with my interactions with my friends. | Strongly Disagree  4 | Disagree  3 | Agree  2 | Strongly Agree  1 |
| 14. During and after my perinatal loss, I was satisfied with my interactions with my nurses. | Strongly Disagree  4 | Disagree  3 | Agree  2 | Strongly Agree  1 |

**Subtotal =_______**

**Table S9: Sensitivity Analysis - Single Variable Regression**

| **Continuous variables** | | |  |
| --- | --- | --- | --- |
| Variable | Coefficient (95% CI) | P-value |  |
|  |  |  |  |
| **Age (10 years)** | -0.046 (-0.109, 0.017) | 0.154 |  |
| **Years schooling** | 0.011 (-0.002, 0.025) | 0.095 |  |
| **Weeks pregnant** | 0.006 (-0.003, 0.015) | 0.167 |  |
| **Previous pregnancies** | -0.028 (-0.056, -0.000) | 0.047 |  |
| **Living children** | -0.021 (-0.055, 0.012) | 0.215 |  |
| **Time since loss (months)** | -0.002 (-0.008, 0.005) | 0.645 |  |
| **Categorical variables** |  |  |  |
| **Variable** |  | **Coefficient (95% CI)** | **P-value** |
|  |  |  |  |
| **Country** | **Kenya** | - | 0.004 |
|  | **Uganda** | 0.130 (0.042, 0.218) |  |
| **Religion** | **Christian** | - | 0.637 |
|  | **Muslim** | 0.034 (-0.108, 0.177) |  |
| **Marital status** | **Living with partner** | - | 0.851 |
|  | **Married** | -0.080 (-0.337, 0.177) |  |
|  | **Widowed** | 0.030 (-0.112, 0.171) |  |
|  | **Separated/divorced** | -0.090 (-0.646, 0.467) |  |
|  | **Prefer not to say** | 0.061 (-0.161, 0.284) |  |
| **Employment** | **Full time** | - | 0.442 |
|  | **Part time** | -0.089 (-0.222, 0.045) |  |
|  | **Unemployed** | -0.082 (-0.194, 0.031) |  |
|  | **Homemaker** | -0.054 (-0.185, 0.078) |  |
| **Type of death** | **Antenatal stillbirth** | - | 0.746 |
|  | **Intrapartum stillbirth** | -0.006 (-0.124, 0.113) |  |
|  | **Neonatal death** | 0.033 (-0.075, 0.141) |  |
| **Sex** | **Male** | - | 0.446 |
|  | **Female** | -0.002 (-0.008, 0.003) |  |
| **Type of birth** | **Normal birth / Vacuum / forceps** | - | 0.131 |
|  | **C-section** | 0.088 (-0.026, 0.202) |  |
| **Pregnancy complications** | **No** | - | 0.228 |
|  | **Yes** | 0.075 (-0.047, 0.197) |  |
| **Previous stillbirths/neonatal deaths** | **No** | - | 0.243 |
|  | **Yes** | -0.073 (-0.195, 0.049) |  |

**Figure S1:** Responses by subscale and item percentage for women in Kenya

**Figure S2:** Responses by subscale and item percentage for women in Uganda.
